# Supplementary material for: Mediterranean Diet Pyramid: A Proposal for Italian People. A Systematic Review of Prospective Studies to Derive Serving Sizes
Source: Nutrients. 2019 Jun 7;11(6):1296. doi: 10.3390/nu11061296 (PMC6628543; doi:10.3390/nu11061296)
Supplement: Supplementary file 1 [file nutrients-11-01296-s001.pdf]

Supplementary Table S1. Weekly menu plan based on advice of Mediterranean Diet Pyramid for Italian People

MONDAY	TUESDAY	WEDNESDAY	THURSDAY	FRIDAY	SATURDAY	SUNDAY
**Breakfast:**<br>-40 g whole wheat flakes<br>-200 g "coscia" pear<br>-30 g walnuts<br>-50 ml semi-skimmed milk	**Breakfast**:<br>-80 g wholemeal sourdough bread<br>-140 g "Italia" grapes<br>-30 g almonds<br>-100 ml semi-skimmed milk 46.25	**Breakfast:**<br>-40 g oat flakes<br>-200 g "percoco" peaches<br>-30 g pistachios<br>-50 g yogurt	**Breakfast:**<br>-80 g wholemeal sourdough bread<br>-200 g "rusticana" prunes<br>-30 g pumpkin seeds<br>- 100 g yogurt 42	**Breakfast:**<br>-40 g oatmeal<br>-260 g "nettarine" peaches<br>-30 g almonds<br>-50 ml semi-skimmed milk	**Breakfast:**<br>-80 g wholemeal sourdough bread<br>-140 g figs<br>-30 g of hazelnuts<br>- 100 ml semi-skimmed milk	**Breakfast:**<br>-40 g whole wheat flakes<br>-200 g "cantalupo" melon<br>-100 g yogurt 42
**Snack:**<br>-100 g fennels<br>-100 g celery	**Snack:**<br>-200 g carrots	**Snack:**<br>-200 g carousels	**Snack:**<br>100 g fennels<br>100 radishes	**Snack:**<br>-200 g of cucumbers	**Snack:**<br>-100 g radishes<br>-100 g raw chicory	**Snack:**<br>-30 g pistachios
**Lunch**:<br>-60 g wholemeal pasta<br>-100 g turnip greens<br>-100 g fresh borlotti beans<br>-100 g "pachino" tomatoes<br>-60 g white bread<br>-20 g extra virgin olive oil<br>-200 g "gala" apple	**Lunch**:<br>-60 g wholemeal pasta<br>-100 g green beans<br>-50 g lentils<br>-100 g mackerel<br>-100 g zucchini<br>-20 g extra virgin olive oil<br>-200 g "agostana" prunes	**Lunch:**<br>-60 g brown rice<br>-50 g chickpeas<br>-100 g green beans<br>-100 g aubergines<br>-30 g sheep cheese<br>-20 g extra virgin olive oil<br>-260 g apricots	**Lunch:**<br>-60 g brown rice<br>-200 g red beetroot<br>- 50 g dried pulses soup<br>-20 g extra virgin olive oil<br>-60 g white bread<br>-200 g "retato" melon	**Lunch:**<br>-60 g barley<br>-50 g lentils<br>-100 g lettuce<br>-100 g rocket salad<br>-20 g extra virgin olive oil<br>-30 g goat cheese<br>-260 g apricots	**Lunch:**<br>-60 g spelled<br>-50 g cannellini beans<br>-100 g lettuce<br>-100 g rocket salad<br>-20 g extra virgin olive oil<br>-200 g "gala" apple	**Lunch:**<br>-60 g white pasta<br>-100 g "ciliegino" tomatoes<br>-100 g aubergines<br>-20 g extra virgin olive oil<br>-200 g beef<br>-80 g wholemeal sourdough bread<br>-100 g lettuce<br>-200 g "agostana" prunes<br>-50 g apple pie
**Dinner**:<br>-80 g wholemeal	**Dinner**:<br>-80 g wholemeal	**Dinner:**<br>-80 g wholemeal	**Dinner:**<br>-80 g wholemeal	**Dinner:**<br>-80 g wholemeal	**Dinner:**<br>-80 g wholemeal	**Dinner:**<br>-80 g wholemeal

sourdough bread<br>-100 g lettuce<br>-100 g chicory<br>-30 g parmigiano<br>-10 g extra virgin olive oil<br>-100 g sardines<br>-200 g "cantalupo" melon	sourdough bread<br>-100 g potatoes<br>-200 g spinach<br>-10 g extra virgin olive oil<br>-1 egg<br>-260 g "nettarine" peaches	sourdough bread<br>-100 g peppers<br>-100 g courgettes<br>-10 g extra virgin olive oil<br>-100 g chicken<br>-100 g potatoes<br>-140 g figs	sourdough bread<br>-100 g "fiaschetto" tomatoes<br>-100 g red onion<br>-10 g extra virgin olive oil<br>- 100 g anchovies<br>-140 g "Italia" grapes	sourdough bread<br>-100 g courgettes<br>-100 g chicken<br>-100 g watercress<br>-10 g extra virgin olive oil<br>-100 g potatoes<br>-140 g prickly pear	sourdough bread<br>-10 g extra virgin olive oil<br>-1 egg<br>-100 g leeks<br>-100 g spinach<br>-200 g "coscia" pears	sourdough bread<br>- 50 g dried fava beans<br>-50 g Parma ham<br>-100 g "insalataro" tomatoes<br>-100 g carousels<br>-100 g radishes<br>-10 g extra virgin olive oil<br>-140 g figs
-1 glass of red wine (140 g)	-1 glass of red wine (140 g)	-1 glass of red wine (140 g)	-1 glass of red wine (140 g)	-1 glass of red wine (140 g)	-1 glass of red wine (140 g)	-1 glass of red wine (140 g)
